# Frequency of Subclinical Atherosclerosis in HIV-infected Brazilians

**DOI:** 10.5935/abc.20180082

**Published:** 2018-05

**Authors:** David Everson Uip

**Affiliations:** Faculdade de Medicina da Universidade de São Paulo (USP), São Paulo, SP - Brazil

**Keywords:** Cardiovascular Diseases, Acquired Immunodeficiency Syndrome, Atherosclerosis, Carotid Intima Media-Carotideo, Vascular Stiffness, Risk Factors

The advances in the treatment of the Human Immunodeficiency Virus (HIV) infection have resulted in a significant reduction in the mortality related to the acquired immunodeficiency syndrome (AIDS). Most patients get infected between the ages of 19 and 39 years, receive medicines from the time of diagnosis onward, with no perspective of interruption. In the follow-up of those patients, chronic non-infectious diseases related to several risk factors, such as age and cardiovascular disease, emerge. Some studies have shown the direct action of the HIV on the vascular endothelium (chronic inflammatory process), in addition to the action of the antiretroviral therapy (ART) on the lipid metabolism.^1^

The incidence of cardiovascular events among HIV-infected patients is low, being, thus, difficult to assess. Subclinical atherosclerosis is associated with an increased risk for events in the general population. It can be detected by use of non-invasive methods, such as carotid ultrasound, aimed at measuring the intimal medial thickness and at assessing the presence of atherosclerotic plaque, in addition to coronary computed tomography to calculate the calcium score. Coronary tomography angiography allows the assessment of the presence, composition and extension of coronary plaques, in addition to detecting stenosis.

The study “*Frequency of Subclinical Atherosclerosis in HIV-Infected Brazilians*”,^2^ aimed at assessing those risk factors for cardiovascular disease, has reported results similar to those of other studies performed in several research centers around the world. It is worth noting the *Multicenter AIDS Cohort Study* (MACS) as a reference.^3^

The MACS is an ongoing prospective study that follows up HIV-infected and non-infected men who have sex with other men in four North American cities (Baltimore/Washington DC, Chicago, Los Angeles and Pittsburgh). The inclusion of cases began in 1987-1991, with new inclusions in 2001-2003, and from 2010 onward. Patients undergo two annual interviews, which include questions about behavior, physical exam and specific and non-specific laboratory tests. From January 2010 to August 2013, 1001 men underwent cardiac computed tomography, 618 of whom were HIV-infected, had ages ranging from 40 and 70 years, and no previous history of myocardial revascularization. That study concluded that coronary plaques, mainly non-calcified ones, were more prevalent and extensive in seropositive patients, regardless of the presence of other risk factors.

Some facts are worth noting: 1. The current increase in the number of HIV-infection cases among young men having sex with other men; 2. The World Health Organization’s recommendation to begin specific therapy as soon as the etiological diagnosis is established; 3. The increased survival of the patients, with a decrease in the occurrence of opportunistic infections; 4. The relevance of the adverse effects presumably caused by ART, mainly osteonecrosis, metabolic syndrome and cardiovascular diseases.

The study in question contradicts the initial view relating atherosclerotic disease to ART and shows the importance of adopting preventive measures regarding the need for a healthy diet, physical exercise practice and the early introduction of medicines to correct the metabolic changes.
